# Healthcare systems data in the context of clinical trials − A comparison of cardiovascular data from a clinical trial dataset with routinely collected data

**DOI:** 10.1016/j.cct.2023.107162

**Published:** 2023-03-16

**Authors:** Archie Macnair, Matthew Nankivell, Macey L. Murray, Stuart D. Rosen, Sally Appleyard, Matthew R. Sydes, Sylvia Forcat, Andrew Welland, Noel W. Clarke, Stephen Mangar, Howard Kynaston, Roger Kockelbergh, Abdulla Al-Hasso, John Deighan, John Marshall, Mahesh Parmar, Ruth E. Langley, Duncan C. Gilbert

**Affiliations:** a MRC Clinical Trials Unit at UCL, Institute of Clinical Trials and Methodology, 90 High Holborn, London WC1V 6LJ, UK; b Health Data Research, UK; c Guys and St Thomas’ NHS Foundation Trust, London, UK; d NHS DigiTrials, NHS Digital, 7 and 8 Wellington Place, Leeds, West Yorkshire LS1 4AP, UK; e National Heart and Lung Institute, Imperial College, London, UK; f University Hospitals Sussex NHS Foundation Trust, Royal Sussex County Hospital, Eastern Road, Brighton BN2 5BE, UK; g BHF Data Science Centre, Health Data Research UK (Central Office), Gibbs Building, 215 Euston Road, London NW1 2BE, UK; h The Christie and Salford Royal Hospitals, Manchester, UK; i Charing Cross Hospital, Imperial College Healthcare NHS Trust, London, UK; j Division of Cancer and Genetics, Cardiff University Medical School, Cardiff, UK; k Department of Urology, University Hospitals of Leicester, Leicester, UK; l Beatson West of Scotland Cancer Centre, Glasgow, UK; m Patient representative, MRC Clinical Trials Unit at UCL, Institute of Clinical Trials and Methodology, 90 High Holborn, London WC1V 6LJ, UK

**Keywords:** Healthcare systems data, Clinical trials, Cardiovascular disease, Prostate cancer

## Abstract

**Background:**

Routinely-collected healthcare systems data (HSD) are proposed to improve the efficiency of clinical trials. A comparison was undertaken between cardiovascular (CVS) data from a clinical trial database with two HSD resources.

**Methods:**

Protocol-defined and clinically reviewed CVS events (heart failure (HF), acute coronary syndrome (ACS), thromboembolic stroke, venous and arterial thromboembolism) were identified within the trial data. Data (using pre-specified codes) was obtained from NHS Hospital Episode Statistics (HES) and National Institute for Cardiovascular Outcomes Research (NICOR) HF and myocardial ischaemia audits for trial participants recruited in England between 2010 and 2018 who had provided consent. The primary comparison was trial data versus HES inpatient (APC) main diagnosis (Box-1). Correlations are presented with descriptive statistics and Venn diagrams. Reasons for non-correlation were explored.

**Results:**

From 1200 eligible participants, 71 protocol-defined clinically reviewed CVS events were recorded in the trial database. 45 resulted in a hospital admission and therefore could have been recorded by either HES APC/NICOR. Of these, 27/45 (60%) were recorded by HES inpatient (Box-1) with an additional 30 potential events also identified. HF and ACS were potentially recorded in all 3 datasets; trial data recorded 18, HES APC 29 and NICOR 24 events respectively. 12/18 (67%) of the HF/ACS events in the trial dataset were recorded by NICOR.

**Conclusion:**

Concordance between datasets was lower than anticipated and the HSD used could not straightforwardly replace current trial practices, nor directly identify protocol-defined CVS events. Further work is required to improve the quality of HSD and consider event definitions when designing clinical trials incorporating HSD.

## 1 Introduction

Over the past two decades, the recording of health management and outcomes in the UK has undergone a gradual transformation with the introduction of digital platforms broadly termed healthcare systems data (HSD). These range from databases that aggregate hospital activity (primarily to facilitate reimbursement) through national treatment datasets (e.g. for chemotherapy or radiotherapy as part of public health repositories) to fully functioning individual electronic patient records (EPR) [1].

As healthcare providers move towards more complete electronic data systems, the opportunity arises to interrogate these directly and obtain data for clinical trial work [2]. Clinical trials underpin evidence-based medicine but are often long-term endeavours with major expense. HSD has been proposed as an alternative source from which to identify potential participants, streamline and improve the efficiency of data collection and significantly reduce costs and time [3]. Challenges in using HSD for clinical trials work include the length of time and permissions required to access to the data, and whether the data provided has the level of detail and accuracy to address the clinical question under investigation within the trial [4,5].

Traditionally the collection of participant data such as treatment received, toxicity and outcomes within a clinical trial, are provided by site-based teams who complete case report forms (CRFs) from individual patients’ hospital notes/GP records and forward to a co-ordinating unit. Additionally, in many clinical trials, endpoint review or adjudication, particularly for cardiovascular (CVS) diagnoses is incorporated into the trial processes to improve the robustness and accuracy of the data.

The recording of CVS events within HSD in comparison to trial data has been evaluated previously. In an early study [6], data from the West of Scotland Coronary Prevention Study was compared to Scottish morbidity records and hospital admissions between 1988 and 1995. There was a strong concordance between datasets (97% for fatal cardiovascular events and over 80% for non-fatal myocardial infarction). However, when stroke and transient ischaemic attack (TIA) events were reviewed, concordance between the trial and routinely collected data was lower at 78%. In contrast, a more recent study of over 21,000 patients which compared the recording of myocardial infarction between 2003 and 2009 in four different HSD sources (a general practice research database, NHS hospital episode statistics (HES), a national myocardial ischaemia audit, and the Office for National Statistics mortality register) concluded that each data source missed a substantial proportion (25−50%) of myocardial infarction events [7].

In an ongoing prostate cancer trial where CVS outcomes were of particular interest and pre-defined in the protocol, we hypothesized that HSD might be able to provide relevant CVS data and improve the efficiency of the trial. To test this hypothesis, we obtained data from two HSD sources: i) NHS Digital requesting HES data covering inpatient stays and accident and emergency attendances (HES APC) and ii) the National Institute for Cardiovascular Outcomes Research (NICOR) heart failure and myocardial ischaemia audits. The aim was to provide a contemporary evaluation of concordance between trial data and current HSD datasets, and to assess whether collecting information about CVS events through HSD in the future would be practical for this trial.

## 2 Methods

### 2.1 Patient population

Eligible participants for this sub-study were those enrolled in England in the main prostate cancer trial between January 2010 and January 2018 who had provided specific consent to allow their data to be accessed from national registries. These parameters were set for the following reasons: i) it became mandatory in 2010 for clinical trial participants in the UK to be asked to provide specific consent for this purpose; ii) national HSD are devolved within the UK and HES APC data is only available in England; and iii) when the study was devised, the most up to date data available through NICOR was for events up to and including January 2018. Ethical approval for this sub-study was included in the main trial protocol.

### 2.2 Dataset definitions

Three data sources were compared. Firstly, the clinical trial dataset where CVS events had been previously identified according to pre-defined protocol definitions (developed with cardiologists − Table 1) and then clinically reviewed, supplemented by additional source data from sites, if required. The specific events of interest were heart failure, acute coronary syndrome, thromboembolic stroke including transient ischaemic episodes, arterial, and venous thromboembolic events (VTE). They were initially identified by site-based staff in oncology and urology departments who completed trial specific case report forms (CRFs) and serious adverse event (SAE) forms. Endpoint review processes within the trial included an initial broad/inclusive approach whereby events covering any cardiological diagnosis or potential cardiological symptoms were reviewed. Approximately 50% of the potential events reported on CRFs and SAE forms did not subsequently meet the specific protocol-defined definitions. These included non-cardiac chest pain, stable angina, or investigation for a silent myocardial infarction that was not confirmed; symptoms that might indicate congestive cardiac failure or VTE (dyspnoea or leg swelling), where investigations did not confirm the diagnosis or symptoms were attributed to another cause; other cardiac events (including atrial fibrillation, hypotension, hypertension, collapse, valve disease, and non-embolic peripheral vascular disease); possible intracerebral bleed, acute transient ischaemic attack, or stroke that was not confirmed on imaging or associated history; and death that on clinical review had sufficient evidence for a non-cardiovascular cause (e.g. malignancy); and other medical events.

The second dataset comprised HES admitted patient care (APC) and accident and emergency (A&E) data from NHS Digital. Although HES APC data at the time of the study also included outpatient and critical care datasets, the diagnostic coding processes for these were considered less reliable and they were not included in this analysis [8]. HES APC data is coded using the 10th version of the International Classification of Diseases (ICD) codes with a main diagnosis in Box-1 and up to 20 other diagnoses. Specific diagnostic codes were used within HES APC to define the events to be compared and these can be accessed in the supplementary material.

The third dataset was the NICOR heart failure and MINAP audits [9,10]. The heart failure audit includes all patients with an unscheduled admission to hospital in England and Wales discharged with a primary diagnosis of heart failure based on the ICD-10 codes available in the supplementary material. MINAP is a national audit of all patients admitted to hospital with an acute coronary syndrome (ACS), as defined as myocardial infarction (MI) whether ST segment elevation or non-ST elevation. Further information of the definition and ICD-10 codes is available in the supplementary material. Submitting data to NICOR is mandatory for UK hospital trusts and linked to reimbursement processes.

### 2.3 Data access, processing and governance

Separate applications were submitted to both NHS Digital and NICOR for access to data, as described previously [4]. Briefly, clinical trial participants were identified to NHS Digital and NICOR using NHS number, name, date of birth, gender and postcode from a secure data-base separate from trial data. Relevant participant data were returned, from NHS Digital and NICOR separately, pseudonymised by study ID as per requirements set out in the relevant regulations and stored in the and held in the UCL Data Safe Haven (DSH). Matching data from NHS Digital and HES APC was returned pseudonymised by study identification number as per requirements set out in the relevant regulations and stored in the DSH. Trial team members had no access to identifiable data. Only specified members of UCL staff with information governance and DSH training could access the NHS Digital and NICOR data.

### 2.4 Analyses

The primary comparison was the trial dataset (used as the reference) versus HES APC (Box-1) diagnosis for each of the five CVS outcomes of interest. Although potentially available, diagnosis boxes 1−20 were not always complete but 50% patients had entries in at least boxes 1−5 and hence a pragmatic decision was taken to use boxes 1−5 in the expanded analyses (see supplementary material for codes). The trial and HES APC data were then also compared with comparable NICOR data if available. Events were matched on date +/− 2 week*s*. Multiple records for the same event were combined into a single data point. For the secondary analyses, HES APC data was extended to include A&E data utilising the main diagnosis Box-1 and using A&E diagnosis coding as defined in the supplementary material. Concordance of event data was presented in tabulated form (correlation coefficients were not used as data was compared at the individual event level). Venn diagrams were created using Stata for the triangulation of the three datasets and positive predictive value/sensitivity calculations assuming the trial dataset as the standard.

NICOR and HES APC data were also reviewed against cardiac events recorded within the trial database that had not met the trial CVS definitions to try to understand any discrepancies. Events were reviewed manually (AM) to describe any common themes or explanations for discrepancies. The trial is ongoing so no data by allocated treatment group are disclosed.

## 3 Results

### 3.1 Cardiovascular events in the clinical trial data, HES APC and NICOR datasets

The eligible study cohort comprised 1200 patients enrolled in a prostate cancer clinical trial in England between January 2010 and January 2018. A total of 71 protocol-defined and clinically reviewed CVS events had previously been identified in this cohort on the trial-specific data collection using CRFs. These consisted of 15 episodes of heart failure, 18 ACS, 17 thromboembolic strokes (including TIAs), 19 VTE and 2 arterial emboli.

The specific CVS events were considered major/significant clinical events and it was expected that the majority would result in a hospital admission. However, on review, 26 out of the 71 trial-reported events occurred entirely outside hospital so would not have been collected by either HES APC nor NICOR data. They were notified to the trials unit as events of particular interest, as opposed to the usual serious adverse event reporting (SAE) that captures hospital admission. These events were either managed in the community or outpatient setting (6 episodes of heart failure, 6 thromboembolic strokes, 5 VTEs and 4 ACS) or were out of hospital (sudden) deaths (*n* = 5; 2 attributed to heart failure and 3 to acute coronary events). The 4 ACS events that did not result in admission were patients who presented to rapid access chest pain clinics with convincing clinical histories of recent myocardial infarction with confirmatory ECG/troponin changes and subsequent (outpatient) coronary angiography. These were then excluded for the subsequent comparison, leaving a total of 45/71 (63%) of the events: 7 episodes of heart failure, 11 ACS, 11 thromboembolic strokes, 14 VTE and 2 arterial emboli.

**Fig. 1:**
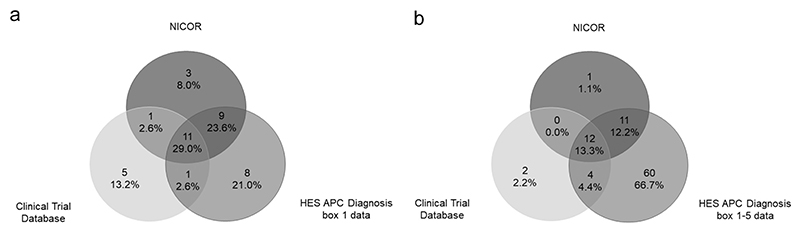
Three-way comparison between clinical trial data, HES APC and NICOR data for the combined outcomes of inpatient ACS and Heart Failure; a) using HES box 1 data only and b) using HES boxes 1−5; 1a − Clinical Trial Data, NICOR and HES (APC box 1 only) ACS and Heart Failure. 1b − Clinical Trial Data, NICOR and HES (APC boxes 1−5) ACS and Heart Failure.

Table 2 shows the total number of events identified in the 3 datasets with Table 3 illustrating the relationship between the events identified in the trial dataset and HES APC. Only 27/45 of events were identified in both the trial dataset and HES Box-1. This translates to a positive predictive value (PPV) of 0.47 and a sensitivity (the proportion of clinical trial data detected within the HES dataset) of 0.60. Widening the HES APC criteria to include diagnosis box 1−5 increased the total number of events identified and the number identified in both datasets to 35/45 resulting in increased sensitivity 0.78 but a fall in the PPV to 0.26. Considering these results in the context of ‘missing data’ (i.e. data not detected in HSD divided by the sum of data detected in HSD plus additional events only detected in the clinical trial dataset), viewing the trial dataset as the primary data and using a tight definition (Box-1) resulted in a missing data rate of 40%. Using data contained within boxes 1−5 reduced this to 22%, albeit with a significantly increased number of events detected in HES that weren’t contained within the trial data. The majority of the extra events identified using HES diagnosis box 1−5 were heart failure events. This is perhaps to be expected as heart failure is a chronic condition potentially recorded over a prolonged time period and during multiple hospital admissions. Only heart failure and ACS were potentially recorded in all 3 datasets. The trial data set had 18 confirmed events, HES Box-1 recorded 29 and NICOR 24. The extent of overlap for the 3 datasets is shown in Fig. 1a. Only 11 events were identified in all 3 datasets and there was a lack of concordance between the 2 HSD’s. Widening the HES APC criteria to include Box 1−5 (Fig. 1b) made little difference to the overall concordance between the 3 datasets but increased the number of heart failure events as described previously. Rates of missing data (pertaining to trial data not detectable in either HSD) reduced from 28% using HES Box-1 to just 11% when Boxes 1−5 were used, although again at a cost of a significant number of additional events that were not seen in the trial data.

To better understand the differences observed between the clinical trial dataset and HES APC data, the 30 potential additional events identified through HES Box-1 diagnoses were reviewed and compared to the trial dataset. Five of these events had been previously identified as part of the CVS endpoint review processes described above but had not met the protocol-defined definition of a CVS event. They included 2 potential heart failure diagnoses, 1 ACS and 2 other events. In addition, of the 9 ACS/heart failure events which were identified by both NICOR and HES Box-1 diagnosis 1 had been reviewed and did not meet the pre-specified criteria. 8 had no record that any event was reported to the trial team.

### 3.2 HES A&E data

HES A&E data for the corresponding patients and time period contained 4362 records of attendance. However, 1562 (38%) of the data either had no codes or were coded ‘diagnosis non classifiable’. Only 561 (13%) contained A&E data dictionary 6-digit coding and just 60 (1%) used ICD-10 codes. Review of the 621 coded events yielded 100 potential CVS events within the defined period. Eleven matched protocol-defined CVS events identified within the trial, accounting for 3 of the 15 heart failure events, 4 of the 18 ACS, 3 of17 cerebrovascular events, and 1/19 venous thromboembolisms. A single event only from the trial dataset that was not detected in HES APC was identified in the A&E data, namely a right leg DVT coded appropriately.

## 4 Discussion

In this study, CVS events that met a pre-defined clinical trial definition (and had been subject to clinical review with, if required, additional source data verification), were compared to data requested from two routinely collected sets of HSD. Agreement between the three datasets was lower than was initially anticipated. There are several potential explanations for this. Firstly, more events than originally anticipated did not result in a hospital admission and therefore would not have been recorded in the two HSD sources accessed. Although, the CVS events identified in the protocol would generally be considered significant/major health events with the majority resulting in a hospital admission it is pertinent that clinical practice is evolving partly due to increasing pressures within the NHS. For example, treatments such as initiating anti-coagulation for asymptomatic venous thromboembolic events identified on routine imaging, a not uncommon event in malignancy, may be managed through outpatient clinics or GP clinics and are therefore not recorded in HES APC or A&E data [11,12].

Secondly, CVS diagnoses often require further investigation or clarification. This is illustrated by both the frequent use of endpoint review committees for CVS trials [13] and the ongoing review of the trial data in this example, where a significant proportion of potential CVS events after subsequent investigations or clinical review were not deemed to meet the trial definitions.

Finally, the recording of clinical details/coding of events within HSD is likely to need more rigour and clarification if they are to be used routinely in a broader context particularly for research. For example, it would be common for patients to be admitted to hospital with a provisional or working diagnosis, with confirmatory tests arranged after discharge where clinically appropriate. This may partly explain our findings that most events were seen in the HES APC data, then NICOR and finally the trial dataset after clinical review/adjudication.

Our findings are in keeping with previous studies where correlation between HSD sources including the two accessed for this study showed a similar lack of concordance for CVS events [7]. In a study of over 20,000 patients it was found that of the patients with non-fatal myocardial infarction, only 31% (95% CI 30.3%−31.6%) were recorded in all 3 datasets (HES APC, NICOR ischaemia audit and the GP clinical practice research datalink (CPRD)) and 64% (95% CI 63.2%−64.6%) were in at least 2 data sources. This study concluded that each data source missed a substantial proportion (25−50%) of myocardial infarction events and that HSD need to be linked to provide an accurate estimation of CVS incidence and outcomes.

In a study comparable to ours, serious vascular events adjudicated from a trial dataset of diabetic patients were compared to HES APC and comparative datasets from Scotland and Wales [14]. Only 1009 of 1401 trial-adjudicated events (72%, 95% CI 69.7%−74.4%) were found in the routine data. Interestingly, the trial result was not meaningfully altered if the routine data was used to calculate the result rather than the trial data set. The relative risk of any serious vascular event or revascularization was 0.88 (95% CI 0.80−0.97) for those receiving the intervention calculated from the adjudicated trial dataset versus 0.90 (95% CI 0.81−0.99) if the routine dataset was used. Though given the modest treatment effect and the width of the CI in this example these results highlight a potential risk of using alternative data sources. The authors speculate that if their study had been designed to use only routinely-collected death and hospitalization data, they estimate that a further 3500 participants would have to have been recruited to maintain the same power and length of follow-up but that despite this, this would still have been a cost-effective approach overall. Of particular relevance to our study is that the authors found that the sensitivity of routine data was lower for ischaemic strokes and particularly transient ischaemic attacks (TIAs) compared to other vascular events and they did not recommend that these events could be collected from routine data sources. They also found, in line with our study that there were events that occurred in the routine datasets that were not present in the trial dataset. All of the potential events in our study that were identified only in the HSD occurred after a minimum follow up period of 3.6 years i.e. later in the patient’s journey, when trial follow up is less frequent.

In another large trial evaluating different cancer screening strategies the completeness and accuracy of routinely collected healthcare data was compared to the trial dataset [15]. The relevant cancer diagnoses (ovarian, tubal or peritoneal) were identified within national cancer registries with a sensitivity of 85% (1125/1324 events identified 95% CI 83.7 to 86.2%). If multiple routinely-collected resources were used the sensitivity increased to 91.1% (1206/1324 events, 95% CI 89.4−92.5%) and there was higher concordance for mortality data. They concluded however that “central adjudication by experts though resource intensive adds value by improving the accuracy of the diagnoses”.

Our study had some noteworthy limitations. The number of events of interest was small and may have affected the precision of the estimate of concordance. Our HSD data sets comprised inpatient care only and may therefore have missed events that were recorded in outpatient notes or GP records, and in addition the defined date of diagnosis window +/− 2 weeks could have been a factor in some instances. For HES APC data, Box-1 and 2 diagnoses may have been a better choice of comparator than either Box-1 alone or Boxes 1−5 inclusive as used in the large diabetic trial described above that considered primary and secondary diagnoses [14]. CVS events within this study were limited to the those as initially defined by the trial protocol; more recent work would likely include a longer list of terms with a commensurately greater number of events [16].

Although the nomenclature may be the same, what constitutes any given event may depend to an extent on the context and method of data collection. To this point the British Heart Foundation (BHF) Data Science Centre has a project (“SCORE-CVD”) looking to define / re-define cardiovascular outcome measures from EHR data. If the study is confined to a single source of data with sufficiently large number of events, then results may closely mirror those that might be generated from a clinical trial, for example the work of Tanner and colleagues who were able to recapitulate results from RCTs in cardiovascular disease in the general population using HSD [17]. From a clinical trials perspective then there is a need to consider both the type of data being requested from healthcare systems data and its relevance to the clinical question under investigation before deciding whether electronic data can improve the efficiency and reduce the costs of a specific clinical trial. For example, the use of healthcare systems data for the RECOVERY trial (NCT04381936), which tested multiple approaches to improving outcomes from Covid-19 during the global pandemic, was both practical, reduced pressure on front-line staff and provided a framework for longer-term follow-up of a newly-emerging infection [18]. In our example however, there was a particular interest in a small number or subset of clinical events (specifically TIAs and other thromboembolic events) to confirm the underlying trial hypothesis in a group of patients undergoing cancer treatment over a prolonged period of time. The precision and coverage of the datasets investigated appeared insufficient to replace routine-data collection of our definition of pertinent events from sites at the present time.

Accepting that the definitions of events may differ subtly between the datasets, an alternative to viewing HSD as a replacement for clinical trial data would be to incorporate all sources of data, e.g. using events captured by HSD but not the trial to augment the evolving trial data. In this way HSD might bring efficiency in terms of augmenting event rates (assuming the definitions were acceptable with respect to the primary outcome of the work) and shortening the overall duration of a trial.

The depth of HSD available continues to grow with time as more and more healthcare systems are digitized. Greater integration of data then from numerous sources would be expected to improve the veracity of what is available for researchers. As the value of this data is increasingly recognized (and disseminated), priority should also be given to improving accuracy through e.g. better support and training for staff inputting source data and more intuitive data entry platforms.

In summary, HSD undoubtedly has an important contribution to make in the conduct of some clinical trials. Large, long-term and/or pragmatic trials with unequivocal outcome measures or where some degree of clinical imprecision can be incorporated are likely to be the most appropriate. In other circumstances, as with patient care, the value of face-to-face interactions should not be underestimated.

## Supplementary Material

Supplementary data to this article can be found online at https://doi.org/10.1016/j.cct.2023.107162.

Supplementary Material

## Figures and Tables

**Table 1:** Protocol cardiovascular events and definition.

Cardiovascular event	Protocol definition
Heart Failure	Defined as new symptoms or clinical signs consistent with a diagnosis of new or decompensated cardiac failure with supporting evidence from chest X-ray, echocardiogram or rise in BNP
Acute Coronary Syndrome (ACS)	including unstable angina, myocardial infarction (including non-ST elevation myocardial infarction) and defined as new onset cardiac chest pain, confirmed as ischaemic in origin by ECG and/or troponin rise +/− coronary angiography)
Thromboembolic stroke	New neurological symptoms and signs with confirmatory evidence from brain CT or MRI (for transient ischaemic attacks the diagnosis was clinical, with corroborative data from carotid duplex scanning)
Other arterial embolic events	Detected by new clinical symptoms and supporting radiological evidence
Venous thromboembolism (VTE)	VTE confirmed radiologically as a deep vein thrombosis (DVT) or pulmonary embolism (PE) confirmed with CT pulmonary angiogram (CTPA)

**Table 2:** Comparison of cardiovascular events recorded in the clinical trial dataset that met the trial definition and had been clinically reviewed, HES APC and NICOR heart failure and MINAP audits.

CVS Event	Number of events			
	Trial database	HES APC diagnosis box 1	HES APC diagnosis box 1−5	NICOR
Heart Failure	7	14	65	15
Acute Coronary Syndrome (ACS)	11	15	22	9
Thromboembolic Stroke	11	15	18	N/A
Other arterial embolism	2	2	4	N/A
Venous Thromboembolism	14	11	27	N/A
Total	**45**	**57**	**136**	**24**

**Table 3:** Clinical Trial Data vs HES APC data for the cardiovascular outcomes.

	HES APC primary diagnosis only			Diagnosis box 1−5		
	Clinical trial database only	HES APC 1 only	Both	Clinical trial database only	HES APC 1-5 only^4^	Both^4^
Heart Failure	3	10	4	1	59	6
Acute Coronary Syndrome (ACS)	3	7	8	1	12^2^	10
Thromboembolic Stroke	3	7^1^	8	2	_9_1	9
Other arterial embolism	2^3^	2	0	2	4	0
Venous Thromboembolism	7	4	7	4	17	10
**Any event**	**18**	**30**	**27**	**10**	**101**	**35**

1 One event is on CRF, with diagnosis “Other arterial embolism”.

2 One event is on CRF, diagnosis “Heart failure” (this event has both HF and ACS diagnoses within NHS boxes 1−5).

3 One event is on NHS, diagnosis “Stroke”.

4 Five events cover two diagnoses in NHS boxes 1−5, and are listed under both categories.

5 Three are not on a trial CRF − 2 events are ACS (box 1) and HF (box 2−5); 1 event has no diagnosis (box 1) and HF and arterial embolism (box 2−5).

6 1 on a trial CRF − 1 is HF on CRF, and HF (box 1) and ACS (box 2−5.)

## Data Availability

Data will be made available on request.
