# Supplementary material for: Healthcare systems data in the context of clinical trials − A comparison of cardiovascular data from a clinical trial dataset with routinely collected data
Source: Contemp Clin Trials. Author manuscript; Available in PMC 2025 Jan 25. (PMC7617340; doi:10.1016/j.cct.2023.107162)
Supplement: Supplementary Material [file EMS202142-supplement-Supplementary_Material.docx]

**Supplementary Material**

**NICOR dataset**

The **Heart failure** audit event inclusion is defined as all those patients with an unscheduled admission to hospital in England and Wales who are discharged with a primary diagnosis of heart failure. This is also defined on discharge of a primary diagnosis of heart failure based on the ICD-10 codes: (33)

I110 Hypertensive heart disease with (congestive) heart failure

I255 Ischaemic cardiomyopathy

I420 Dilated cardiomyopathy

I429 Cardiomyopathy, unspecified

I500 Congestive heart failure

I501 Left ventricular failure

I509 Heart failure, unspecified

The **MINAP** audit is a national audit of all admissions admitted to hospital with acute coronary syndrome. Over the last two summary reporting years (2018/2019) the MINAP audit has been including in the annual report data verification between HES data and data submitted to the audit to demonstrate hospital variation in reporting based on defined ICD-10 discharge codes. In the last annual report of 2019 at time of writing (years covering 2017-2018) they have defined inclusion into the audit using the following ICD-10 codes as: (34)

STEMI: all patients discharged with final diagnosis of STEMI – identified by the presence of the following ICD 10 codes in ANY position:

I21.0 ST elevation (STEMI) myocardial infarction of anterior wall;

I21.1 ST elevation (STEMI) myocardial infarction of inferior wall;

I21.2 ST elevation (STEMI) myocardial infarction of other sites;

I21.3 ST elevation (STEMI) myocardial infarction of unspecified site.

NSTEMI: all patients discharged with final diagnosis of NSTEMI – identified by the presence of the following code in the FIRST position:

I21.4 Acute subendocardial myocardial infarction.

MINAP would only use events for their annual report that met this criteria on ICD-10 codes. This would only affect a minority of the cases within this comparison and therefore did not affect the decision on which codes to be used in HES APC in this comparison.

**ICD10 codes for analysis- *indicates that all four digit codes used with starting three digits**

| Protocol-defined VS Diagnosis | HES: ICD-10 code version 2019 and corresponding diagnosis with ICD-10 code |
| --- | --- |
| Acute coronary syndrome | I21* Acute myocardial infarction  I22* Subsequent myocardial infarction  I23* Current complications following acute myocardial infarction  I249 Acute ischaemic heart disease, unspecified |
| Heart failure | I110 Hypertensive heart disease with (congestive) heart failure I255 Ischaemic cardiomyopathy I420 Dilated cardiomyopathy I429 Cardiomyopathy, unspecified I500 Congestive heart failure I501 Left ventricular failure I509 Heart failure, unspecified |
| Thromboembolic stroke | I63* Cerebral Infarction  I64* Stroke not specified as haemorrhage or infarction (thromboembolic stroke)  G45* Transient cerebral Ischaemic attacks and related syndromes |
| Venous thromboembolism | I26* Pulmonary Embolism  I802 Phlebitis and thrombophlebitis of other deep vessels of lower extremities incl deep vein thrombosis NOS  1803 Phlebitis and thrombophlebitis of lower extremities, unspecified including embolism or thrombosis of lower extremity NOS  I81* Portal vein thrombosis  I82* Other venous embolism and thrombosis |
| Other arterial embolic event | I74* Arterial embolism and thrombosis |

ICD10 codes for triangulation analysis- *indicates that all four digit codes used with starting three digits

A&E coding for diagnosis uses a six character code with diagnosis condition (2n) sub analysis (1n) anatomical area (2n) and then anatomical side (1n). HES A&E events will be matched with trial cardiovascular event points using the following codes at the start of valid codes as per A/E data dictionary: (37)

• 20; cardiac condition

• 21; Cerebrovascular condition

• 22; Vascular condition

• 201; myocardial infarction

• 202; other cardiac condition would be excluded

Examples of how the coding includes would be:

20122L- 201 (Cardiac conditions - myocardial ischaemia & infarction) 22 (Chest) L (Left) - Myocardial infarction

22832R- 22 (Other vascular condition) 8 (filling character) 32 (leg) R - DVT right leg
